# Comparison of Performance of Psychiatrists vs Other Outpatient Physicians in the 2020 US Medicare Merit-Based Incentive Payment System

**DOI:** 10.1001/jamahealthforum.2022.0212

**Published:** 2022-03-25

**Authors:** Andrew C. Qi, Karen E. Joynt Maddox, Laura J. Bierut, Kenton J. Johnston

**Affiliations:** 1 Department of Medicine, Washington University School of Medicine, St Louis, Missouri; 2 Department of Medicine, Brigham and Women’s Hospital, Boston, Massachusetts; 3 Department of Psychiatry, Washington University School of Medicine, St Louis, Missouri; 4 Department of Health Management and Policy, College for Public Health and Social Justice, St Louis University, St Louis, Missouri

## Abstract

**Importance:**

Medicare’s Merit-Based Incentive Payment System (MIPS) is a new, mandatory, outpatient value-based payment program that ties reimbursement to performance on cost and quality measures for many US clinicians. However, it is currently unknown how the program measures the performance of psychiatrists, who often treat a different patient case mix with different clinical considerations than do other outpatient clinicians.

**Objective:**

To compare performance scores and value-based reimbursement for psychiatrists vs other outpatient physicians in the 2020 MIPS.

**Design, Setting, and Participants:**

In this cross-sectional study, the Centers for Medicare & Medicaid Services Provider Data Catalog was used to identify outpatient Medicare physicians listed in the National Downloadable File between January 1, 2018, and December 31, 2020, who participated in the 2020 MIPS and received a publicly reported final performance score. Data from the 593 863 clinicians participating in the 2020 MIPS were used to compare differences in the 2020 MIPS performance scores and value-based reimbursement (based on performance in 2018) for psychiatrists vs other physicians, adjusting for physician, patient, and practice area characteristics.

**Exposures:**

Participation in MIPS.

**Main Outcomes and Measures:**

Primary outcomes were final MIPS performance score and negative (penalty), positive, and exceptional performance bonus payment adjustments. Secondary outcomes were scores in the MIPS performance domains: quality, promoting interoperability, improvement activities, and cost.

**Results:**

This study included 9356 psychiatrists (3407 [36.4%] female and 5 949 [63.6%] male) and 196 306 other outpatient physicians (69 221 [35.3%] female and 127 085 [64.7%] male) (data on age and race are not available). Compared with other physicians, psychiatrists were less likely to be affiliated with a safety-net hospital (2119 [22.6%] vs 64 997 [33.1%]) or a major teaching hospital (2148 [23.0%] vs 53 321 [27.2%]) and had lower annual Medicare patient volume (181 vs 437 patients) and mean patient risk scores (1.65 vs 1.78) (*P* < .001 for all). The mean final MIPS performance score for psychiatrists was 84.0 vs 89.7 for other physicians (absolute difference, −5.7; 95% CI, −6.2 to −5.2). A total of 573 psychiatrists (6.1%) received a penalty vs 5739 (2.9%) of other physicians (absolute difference, 3.2%; 95% CI, 2.8%-3.6%); 8664 psychiatrists (92.6%) vs 189 037 other physicians (96.3%) received a positive payment adjustment (absolute difference, −3.7%; 95% CI, −3.3% to −4.1%), and 7672 psychiatrists (82.0%) vs 174 040 other physicians (88.7%) received a bonus payment adjustment (absolute difference, −6.7%; 95% CI, −6.0% to −7.3%). These differences remained significant after adjustment.

**Conclusions and Relevance:**

In this cross-sectional study that compared US psychiatrists with other outpatient physicians, psychiatrists had significantly lower 2020 MIPS performance scores, were penalized more frequently, and received fewer bonuses. Policy makers should evaluate whether current MIPS performance measures appropriately assess the performance of psychiatrists.

## Key Points

**Question:**

How did psychiatrists perform in the 2020 Medicare Merit-Based Incentive Payment System (MIPS) compared with other outpatient physicians?

**Findings:**

In this cross-sectional study of 9356 psychiatrists and 196 306 other outpatient physicians participating in the 2020 MIPS, psychiatrists had significantly lower performance scores, were significantly more likely to be assessed a performance penalty, and were less likely to be assessed a bonus than other physicians.

**Meaning:**

Psychiatrists performed worse than other physicians in Medicare’s new mandatory outpatient value-based payment system; therefore, more research is needed to evaluate the appropriateness of MIPS measures for psychiatrists.

## Teaser

This cross-sectional study compares the performance of psychiatrists vs other outpatient physicians in Medicare’s Merit-Based Incentive Payment System.

## Introduction

The Merit-Based Incentive Payment System (MIPS) is a mandatory, value-based payment program implemented by the Centers for Medicare & Medicaid Services (CMS) that covers almost all outpatient US clinicians who treat Medicare patients. In 2020, the second payment year for MIPS, up to 5% of clinicians’ Medicare reimbursement was tied to their 2018 performance across 4 measurement domains: quality, promoting interoperability, improvement activities, and cost.^1^ As the program is phased in, payment penalties and bonuses will increase to up to 9% of clinicians’ Medicare reimbursement by the end of 2022.^2^

The MIPS was designed to assess performance for a broad range of outpatient clinicians, although different clinicians practice in widely disparate settings and elect to report different quality measures. Currently, there are 210 MIPS quality measures from which clinicians must select at least 6 to report to the CMS.^3,4^ This program design may create particular challenges for specialists,^5,6,7,8,9^ especially those who are unlikely to report common measures, such as hypertension or diabetes control. There are few MIPS measures specific to specialist practice, and specialists reporting as part of a multispecialty group or accountable care organization may rely on group reporting of nonspecialty-specific measures.^5,6,10,11^ Furthermore, even for specialties for which specific measures have been developed, these measures may not be usable given the CMS’s plans to phase out measures with broadly high performance.^12^ Questions have also been raised about the adequacy of risk adjustment for the different patient populations seen by different types of specialists.^13^

Psychiatrists represent one group for whom MIPS may be particularly poorly suited to adequately assess care quality. Concerns have been raised that there are relatively few well-defined and widely accepted behavioral health quality measures compared with other medical fields.^14,15^ Although there were 25 measures in the mental/behavioral health specialty set for 2018, all but 3 were listed in sets for other specialties, suggesting a lack of measures specific to the expertise of psychiatrists.^3^ Furthermore, there is currently no adjustment for some of the most prevalent mental health conditions in quality measures, despite evidence that these disorders are associated with higher morbidity, mortality, and cost independent of other medical comorbidities.^16,17^

As financial penalties for nonparticipation or poor performance increase, it is critical to understand the effect of MIPS on psychiatric practice. This study aims to examine 2 key questions. First, how did performance scores and payment adjustments assessed under the 2020 MIPS for psychiatrists compare with those for other outpatient physicians? Second, were there differences in reporting rates or performance scores on specific performance measures that may help explain these differences?

## Methods

This cross-sectional study was deemed exempt by the Saint Louis University Institutional Review Board because the data are publicly available on the World Wide Web and are deidentified; therefore, informed consent was not obtained from the participants. The study followed Strengthening the Reporting of Observational Studies in Epidemiology (STROBE) reporting guideline for cross-sectional studies.

### Study Population

We used the CMS Provider Data Catalog to identify outpatient Medicare physicians listed in the National Downloadable File between January 1, 2018, and December 31, 2020, who participated in the 2020 MIPS and received a publicly reported final performance score.^18^ We included physicians with a listed specialty of primary care, medical specialist, obstetrics-gynecology, or psychiatry, as defined in the Medicare Data on Provider Practice and Specialty.^19^ The MIPS data were linked to the 2018 Medicare physician and other supplier reports using physicians’ National Provider Identifiers (NPIs).

### Covariates

Street addresses of practice locations in the National Downloadable File were geocoded and linked to data from the 2016 American Community Survey, 2018 Area Deprivation Index,^20,21^ 2018 Dartmouth Atlas of Healthcare Hospital Referral Regions, and 2010 US Census tract rural-urban commuting area codes. We excluded physicians missing data on key variables from the primary analyses, although we included them in sensitivity analyses to test the robustness of our findings.

### Outcomes

Our primary outcomes were physicians’ final performance scores reported under the 2020 MIPS based on measured performance during 2018, as well as their receipt of negative (penalty), positive, and exceptional performance bonus payment adjustments. Final performance scores were weighted composite measures derived from 4 domains: quality (eg, diabetes control), cost of care (eg, total per capita costs), promoting interoperability (eg, using a certified electronic medical record), and practice improvement activities (eg, participating in clinical registries). The final performance scores in the default MIPS track were weighted at 50% for quality, 10% for cost, 25% for promoting interoperability, and 15% for improvement activities.^22^

Because some clinicians participated in multiple practices and thus had multiple MIPS performance scores associated with their NPIs, we used a hierarchy developed by the CMS to assign scores: physicians participating in a MIPS alternative payment model (APM) were assigned the highest score received as part of any APM, and physicians not participating in an APM were assigned the highest score received via any other reporting mechanism.^1^ Payment adjustments were identified by applying performance score thresholds established by the CMS for the 2020 MIPS: (1) negative payment adjustment for performance scores less than 15, (2) positive payment adjustment for performance scores greater than 15 (a score of exactly 15 received neither a negative nor a positive payment adjustment), and (3) exceptional bonus payment adjustment for performance scores of 70 or higher.^1^ In 2020, these values represented adjustments between −5% and +1.68% of clinicians’ total Medicare Part B reimbursement.^23^ Secondary outcomes were scores in each of the 4 MIPS performance domains, as well as the top 20 individual MIPS performance measures reported in the study population.

### Physician, Patient, and Practice Area Characteristics

We used the 2018-2020 National Downloadable File and the 2018 Medicare physician and other supplier reports to identify physician sex, number of years since medical school graduation, Medicare patient caseload, and mean patient risk scores on the CMS Hierarchical Condition Categories risk score. Using linked data from the 2018 CMS Impact File, we classified any clinician affiliated with a safety net hospital (top quartile nationally of disproportionate share hospitals) as a safety net clinician and any clinician affiliated with a general acute care hospital with a resident-to-bed ratio of 0.25 or greater as being affiliated with a major teaching hospital. We used geocoded data on clinician practice locations to identify Area Deprivation Index national rank,^21^ rural vs urban location,^24^ US Census region, and Dartmouth hospital referral region.

### Statistical Analysis

We compared physician, patient, and practice area characteristics for psychiatrists vs other outpatient physicians, using the χ^2^ test to test for differences in proportions and the independent sample 2-tailed *t* test for differences in means. We estimated clinician-level multivariable regression models to assess the association between psychiatrist vs other physician specialty and the primary outcomes. We used linear regression for the MIPS final performance score and the 4 domain scores and logistic regression for the binary payment adjustment outcomes (negative, positive, and bonus payment adjustment). For each estimate, we report unadjusted results (reflecting current MIPS methods) as well as results adjusted for the characteristics detailed in Table 1 to control for potential confounders. We also added market-level fixed effects for 306 Dartmouth hospital referral regions to estimate within-market associations. We report adjusted results as marginal differences in dependent variables per unit change in the independent variable.

**Table 1.** Characteristics of Psychiatrists and Other Outpatient Physicians Participating in the 2020 Medicare Merit-Based Incentive Payment System^a^

Characteristic	Psychiatrists (n = 9356)^b^	Other outpatient physicians (n = 196 306)	*P* value
Physician characteristics			
Sex			
Female	3407 (36.4)	69 221 (35.3)	.02
Male	5949 (63.6)	127 085 (64.7)	
Time since medical school graduation, mean (SD), y	24 (13)	23 (12)	<.001
Primary care physician^c^	0	104 736 (53.4)	NA
Specialist^d^	9356 (100)	91 570 (46.6)	
Affiliated with a safety net hospital	2119 (22.6)	64 997 (33.1)	<.001
Affiliated with a major teaching hospital	2148 (23.0)	53 321 (27.2)	<.001
Medicare patient caseload characteristics			
Total Medicare beneficiaries, mean (SD)^e^	181 (219)	437 (490)	<.001
CMS-HCC Risk Score, mean (SD)^f^	1.65 (0.49)	1.78 (0.87)	<.001
Local practice area characteristics^g^			
Area Deprivation Index national rank, mean (SD)^h^	46 (28)	45 (26)	<.001
Rural	1325 (14.2)	31 758 (16.2)	<.001
Urban	8619 (92.1)	177 442 (90.4)	<.001
US Census region			
Northeast	2817 (30.1)	43 042 (21.9)	<.001
South	2896 (31.0)	73 373 (37.4)	
Midwest	2206 (23.6)	43 813 (22.3)	
West	1436 (15.3)	36 072 (18.4)	
Other	0	0	

Abbreviations: CMS, Centers for Medicare & Medicaid Services; HCC, hierarchical condition categories; NA, not applicable.

^a^ Data are presented as number (percentage) of physicians unless otherwise indicated.

^b^ Includes all physicians who listed a primary or secondary specialty of psychiatry, geriatric psychiatry, or neuropsychiatry.

^c^ Includes all physicians who listed a medical specialty of geriatric medicine, internal medicine, family medicine, general practice, obstetrics-gynecology, or pediatric medicine.

^d^ Includes all physicians who were not primary care physicians.

^e^ Seen as patients in 2018 as reported in the Medicare Physician and Other Supplier Reports. Because of extreme outliers in this data set, we winsorized this number before analysis using the entire distribution in the sample, setting the bottom 1% equal to the first percentile value (n = 14) and the top 1% equal to the 99th percentile value (n = 2279).

^f^ Based on patients’ age, sex, original reason for Medicare eligibility, dual Medicaid enrollment, institutionalization in long-term care, and 83 clinical conditions identified by diagnoses in Medicare claims. Higher scores imply sicker and higher-cost patients. The CMS-HCC risk score ranges from 0.43 to 10.07 in our population, with an IQR of 1.15 to 2.19 and a median of 1.55. A mean score of 1.78 implies a patient caseload 78% sicker than the national average.

^g^ Note that many clinicians have multiple practice locations. As a result, the Area Deprivation Index national rank represents the mean for each clinician across all their practice locations. In addition, some clinicians had practice locations in both rural and urban areas, and others had practice locations in more than 1 US Census region; thus, these numbers do not sum to 100%.

^h^ A measure of local neighborhood area socioeconomic disadvantage derived from US Census tract data on income, educational level, employment, and housing quality, the Area Deprivation Index national rank ranges from 0 to 100 and indicates the percentile rank of disadvantage for a given US Census tract. Numbers closer to 100 indicate greater disadvantage. Numbers closer to 50 are indicative of the national mean.

We conducted an exploratory analysis of the 20 most frequently reported MIPS performance measures in the study population. We report descriptive statistics on the percentage of clinicians reporting each measure and their mean performance scores on these measures, comparing psychiatrists with other physicians. On the basis of prior research, we identified measures that may be dependent on technology.^25^

In sensitivity analyses, we compared psychiatrists with other physicians included in our study on additional patient caseload variables for those who had available data in the 2018 Medicare physician and other supplier reports. Lastly, we reestimated the models for our primary outcomes, assessing the same associations among an expanded population of outpatient physicians (adding back those previously excluded for missing data on key variables) that adjusted for fewer variables and among a reduced population with more data available on patient caseload that adjusted for additional variables.

We performed all analyses using SAS statistical software, version 9.4 (SAS Institute Inc) and Stata software, version 15.1 (StataCorp). A 2-sided *P* < .05 was considered to be statistically significant.

## Results

### Study Population

**Figure 1.**
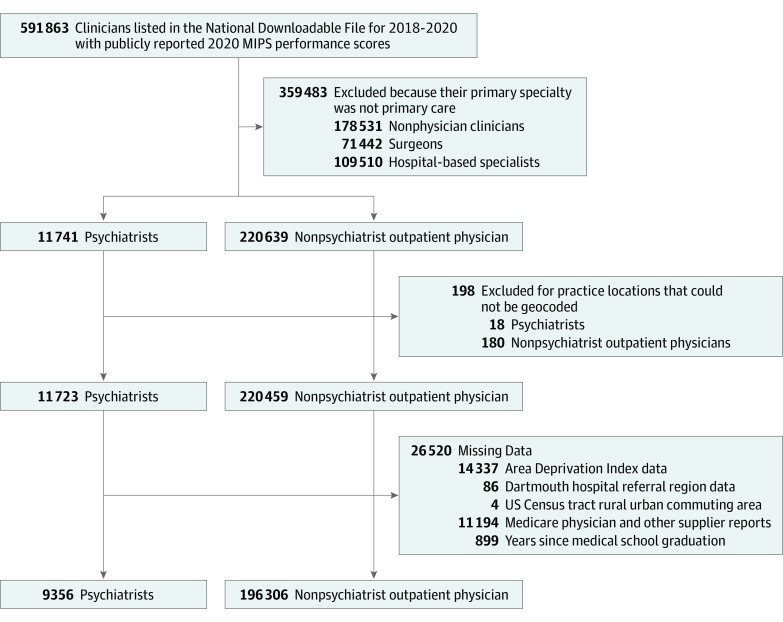
Study Sample Selection Flowchart MIPS indicates Merit-Based Incentive Payment System.

Of the 593 863 clinicians listed in the National Downloadable File in 2018 to 2020 participating in the 2020 MIPS, 359 483 were excluded because their primary specialty was not primary care, a medical specialty, obstetrics-gynecology, or psychiatry (Figure 1). Of the remaining clinicians, we excluded 198 whose practice locations could not be geocoded and 26 520 who were missing data on key variables. Our final study population consisted of 205 662 clinicians, including 9356 psychiatrists (3407 [36.4%] female and 5 949 [63.6%] male) and 196 306 other physicians (69 221 [35.3%] female and 127 085 [64.7%] male) (data on age and race are not available). Compared with physicians included in the study, those excluded were more likely to be psychiatrists (213 [8.9%] vs 426 [4.5%]) or medical specialists (15 151 [56.7%] vs 100 926 [49.1%]) and had a lower mean patient caseload (290 [383] vs 425 [484] patients) (eTable 1 in the Supplement). Psychiatrists were less likely to participate in MIPS in 2018 than other outpatient physicians in the National Downloadable File who billed Medicare during 2018 to 2020 (11 730 [41.1%] vs 221 118 [59.1%]) (eTable 2 in the Supplement).

Psychiatrists were less likely than other physicians to be affiliated with a safety net (2119 [22.6%] vs 64 997 [33.1%]) or major teaching hospital (2148 [23.0%] vs 53 321 [27.2%]) or have a rural practice location (1325 [14.2%] vs 31 758 [16.2%]) and had lower annual Medicare patient caseloads (181 vs 437) and hierarchical condition category scores (1.65 vs 1.78) (Table 1). Psychiatrists also treated patients who had a younger mean (SD) age (59.6 [8.9] vs 71.8 [5.1] years) and saw a markedly higher proportion of patients with diagnosed depression (69.1% vs 29.9%) or dual enrollment in Medicaid (56.6% vs 26.7%). Psychiatrists’ patients were also more often male (45.0% vs 40.7%) and races other than white (27.0% vs 22.3%) (eTable 3 in the Supplement).

### Comparison of Psychiatrists With Other Outpatient Physicians on Overall MIPS Performance

The mean (SD) MIPS final performance score for physicians in our study was 89.4 (23.7), with 6312 (3.1%) receiving negative payment adjustments, 197 700 (96.1%) receiving positive payment adjustments, and 181 712 (88.4%) receiving bonus payment adjustments (eTable 1 in the Supplement). The mean (SD) MIPS final performance score for psychiatrists was lower than other physicians (84.0 [29.7] vs 89.7 [23.3]; absolute difference, −5.7; 95% CI, −6.2 to −5.2) (Table 2). The number of psychiatrists who received a negative payment adjustment was 573 (6.1%) vs 5 739 (2.9%) for other physicians (absolute difference, 3.2%; 95% CI, 2.8%-3.6%); 8664 (92.6%) of psychiatrists vs 189 037 (96.3%) of other physicians received a positive payment adjustment (absolute difference, −3.7%; 95% CI, −4.1% to −3.3%), and 7672 (82.0%) of psychiatrists vs 174 040 (88.7%) of other physicians received a bonus payment adjustment (absolute difference, −6.7%; 95% CI, −7.3% to −6.0%).

**Table 2.** Association of Psychiatry vs Other Outpatient Physician Specialty Type With 2020 Merit-Based Incentive Payment System Performance Scores

Variable	Unadjusted results			Adjusted results^a^	
	Psychiatrists (n = 9356)	Other outpatient physicians (n = 196 306)	Absolute difference (95% CI)	Marginal difference of psychiatry vs other specialty (95% CI)^b^	Relative difference of psychiatry vs other specialty, % (95% CI)^c^
Final performance score, mean (SD)	84.0 (29.7)	89.6 (23.3)	−5.6 (−6.1 to −5.1)	−6.3 (−6.8 to −5.8)	−7.1 (−7.6 to −6.5)
Negative payment adjustment, No. (%)	573 (6.1)	5739 (2.9)	3.2 (2.8 to 3.6)	2.1 (1.8 to 2.5)	69.6 (58.7 to 80.4)
Positive payment adjustment, No. (%)	8663 (92.6)	189 037 (96.3)	−3.7 (−4.1 to −3.3)	−2.9 (−3.3 to −2.5)	−3.1 (−3.5 to −2.6)
Bonus payment adjustment, No. (%)	7672 (82.0)	174 040 (88.7)	−6.7 (−7.3 to −6.0)	−6.9 (−7.7 to −6.2)	−7.9 (−8.7 to −7.0)

^a^ Multivariable regression models were used to estimate the findings, which also adjusted for the physician, patient caseload, and local practice area characteristics listed in Table 1 (with the exception of primary care/specialist status) and with the addition of fixed effects for Dartmouth hospital referral regions. Ordinary least-squares regression was used to model the final performance score outcome and logistic regression to model the 3 binary payment adjustment indicators.

^b^ The marginal differences in the outcome are reported as the change in the mean of the dependent variables associated with a unit change in the independent variables (ie, the marginal effect).

^c^ The relative differences in the outcome are reported as the marginal difference divided by the study population mean.

After adjustment, compared with other physicians, psychiatrists had lower MIPS final performance scores (difference, −6.3; 95% CI, −6.8 to −5.9), higher likelihood of receiving a negative payment adjustment (difference, 2.1%; 95% CI, 1.8% to 2.5%), and lower likelihoods of receiving a positive (difference, −2.9%; 95% CI, −3.3% to −2.5%) or bonus (difference, −6.9%; 95% CI, −7.7% to −6.2%) payment adjustment.

Results were similar in sensitivity analyses of the primary outcomes among an expanded population that included physicians with missing data and among a reduced population of physicians that adjusted for the additional patient caseload variables of dual Medicaid enrollment, age, sex, and race (eTable 4 in the Supplement). The magnitude of differences was greater in the reduced population, although this finding was consistent with or without adjusting for the additional variables.

### Comparison of Psychiatrists With Other Outpatient Physicians on MIPS Domain Score Performance

**Figure 2.**
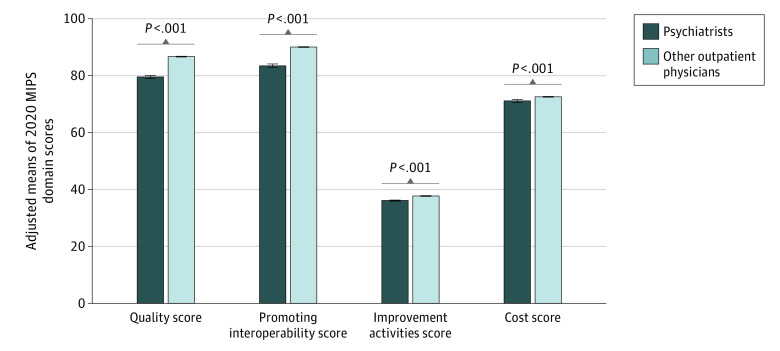
Differences in 2020 Risk-Adjusted US Medicare Merit-Based Incentive Payment System (MIPS) Domain Scores for Psychiatrists vs Other Outpatient Physicians Multivariable linear regression models were used to estimate the adjusted mean scores, adjusting for the individual clinician, patient caseload, and local practice area characteristics listed in Table 1 (excluding primary care/specialist status) and including fixed effects for the Dartmouth hospital referral regions. We report the adjusted means of the scores with Wald testing of significance between psychiatrist and nonpsychiatrist means. Error bars indicate 95% CIs.

In secondary analyses, compared with other physicians, psychiatrists had lower adjusted means across all 4 of the 2020 MIPS domain scores. The largest absolute differences were in the quality (adjusted mean, 79.6 vs 86.7) and promoting interoperability (adjusted mean, 83.5 vs 90.1) domain scores (Figure 2; eTable 5 in the Supplement).

### Exploratory Analysis of the Top 20 Individual MIPS Performance Measures

The top 20 measures reported by the study population are listed in Table 3. The most frequently reported measures were identical among psychiatrists and nonpsychiatrists and were largely unrelated or nonspecific to psychiatric practice. Of the top 20 measures, only 5 are included in the mental/behavioral health specialty set, all of which are also in the specialty sets for internal medicine and family medicine.

**Table 3.** Comparison of the Top 20 Merit-Based Incentive Payment System Performance Measures Publicly Reported for 2020 in the Study Population^a^

Variable	Physicians reporting, No. (%)^b^				Performance scores, mean (SD)^c^		
	All physicians (n = 205 662)	Psychiatrists (n = 9356)	Other outpatient physicians (n = 196 306)	Absolute difference (95% CI)	Psychiatrists	Other outpatient physicians	Absolute difference (95% CI)
Technology-dependent measures							
Provide patient access	145 077 (70.5)	6622 (70.8)	138 455 (70.5)	0.3 (−0.6 to 1.3)	84.0 (21.8)	83.8 (20.5)	0.1 (−0.4 to 0.7)
Electronic prescribing	144 194 (70.1)	6583 (70.4)	137 611 (70.1)	0.3 (−0.7 to 1.2)	90.6 (12.5)	90.8 (12.0)	−0.2 (−0.5 to 0.1)
Secure messaging	141 049 (68.6)	6362 (68.0)	134 687 (68.6)	−0.6 (−1.5 to 0.4)	25.4 (18.8)	27.1 (20.0)	−1.6 (−2.1 to −1.1)
Health information exchange	112 485 (54.7)	4651 (49.7)	107 834 (54.9)	−5.3 (−6.3 to −4.2)	22.8 (22.2)	25.5 (23.8)	−2.8 (−3.5 to −2.1)
View, download, or transmit	67 396 (32.8)	3028 (32.4)	64 368 (32.8)	−0.6 (−1.6 to 0.4)	24.9 (18.2)	26.8 (19.3)	−1.8 (−2.5 to −1.1)
Nontechnology-dependent measures							
Patient-specific educational level	143 440 (69.7)	6461 (69.1)	136 979 (69.8)	−0.7 (−1.6 to 0.3)	71.6 (30.4)	69.0 (31.0)	2.6 (1.9 to 3.4)
Medication reconciliation	120 311 (58.5)	5368 (57.4)	114 943 (58.6)	−1.3 (−2.3 to −0.3)	83.6 (15.6)	86.3 (15.7)	−2.7 (−3.1 to −2.3)
Preventive care and screening: body mass index screening and follow-up plan	54 714 (26.6)	3157 (33.7)	51 557 (26.3)	7.5 (6.6 to 8.5)	50.4 (23.9)	53.3 (24.2)	−2.9 (−3.8 to −2.1)
Pneumococcal vaccination status for older adults	54 622 (26.6)	2759 (29.5)	51 863 (26.4)	3.2 (2.3 to 4.1)	60.4 (22.9)	61.7 (22.2)	−1.3 (−2.2 to −0.5)
Documentation of current medications in the medical record	52 491 (25.5)	2649 (28.3)	49 842 (25.4)	2.9 (2.0 to 3.8)	80.8 (20.1)	89.5 (14.1)	−8.7 (−9.3 to −8.2)
Breast cancer screening	48 132 (23.4)	2553 (27.3)	45 579 (23.2)	4.1 (3.2 to 5.0)	53.7 (19.6)	57.1 (18.4)	−3.4 (−4.1 to −2.7)
Colorectal cancer screening	46 935 (22.8)	2388 (25.5)	44 547 (22.7)	2.9 (2.0 to 3.8)	48.4 (19.7)	51.8 (20.0)	−3.5 (−4.3 to −2.6)
Falls: screening for future fall risk	46 484 (22.6)	2599 (27.8)	43 885 (22.4)	5.5 (4.6 to 6.4)	63.2 (27.3)	58.4 (29.2)	4.8 (3.6 to 5.9)
Preventive care and screening: influenza immunization	44 324 (21.6)	2516 (26.9)	41 808 (21.3)	5.6 (4.8 to 6.5)	45.3 (21.1)	47.1 (21.8)	−1.8 (−2.6 to −0.9)
Ischemic vascular disease: use of aspirin or another antiplatelet	41 741 (20.3)	1991 (21.3)	39 750 (20.2)	1.0 (0.2 to 1.9)	81.0 (8.4)	80.7 (10.5)	0.3 (−0.2 to 0.8)
Preventive care and screening							
Tobacco use: screening and cessation intervention	41 442 (20.2)	2375 (25.4)	39 067 (19.9)	5.5 (4.7 to 6.4)	66.2 (31.8)	67.4 (33.0)	−1.2 (−2.5 to 0.2)
Screening for depression and follow-up plan	38 699 (18.8)	2542 (27.2)	36 157 (18.4)	8.8 (8.0 to 9.6)	43.4 (28.5)	40.9 (27.7)	2.5 (1.4 to 3.6)
Use of high-risk medications in the elderly	33 722 (16.4)	1765 (18.9)	31 957 (16.3)	2.5 (1.8 to 3.3)	8.2 (7.1)	6.1 (6.5)	2.0 (1.7 to 2.3)
Weight assessment and counseling for nutrition and physical activity for children and adolescents	24 739 (12.0)	1555 (16.6)	23 184 (11.8)	4.9 (4.2 to 5.5)	46.6 (36.2)	49.8 (35.8)	−3.4 (−5.2 to −1.6)
Depression utilization of the PHQ-9 tool	18 233 (8.9)	1171 (12.5)	17 062 (8.7)	3.8 (3.2 to 4.4)	26.1 (20.9)	24.8 (17.5)	1.2 (0.1 to 2.2)

Abbreviation: PHQ-9, Patient Health Questionnaire 9.

^a^ This table displays an exploratory analysis of reporting on individual performance measures to explore potential underlying mechanisms of performance score differences between psychiatrists and other outpatient physicians.

^b^ Physicians who met the study inclusion and exclusion criteria and had data publicly reported on individual Merit-Based Incentive Payment System (MIPS) measures. Note that not all physicians included in our study had data publicly reported on individual measures (although they did all have an overall MIPS score and payment adjustment reported).

^c^ Mean performance scores of physicians with publicly reported measure data, as indicated in the number (percentage) reporting. To get the number of physicians reporting for mean performance scores, multiply the percentage reporting by the total number of physicians in the study population for each measure.

Psychiatrists had similar-to-lower reporting rates and performance scores on technology-dependent MIPS performance measures. Psychiatrists were significantly less likely to report participating in a health information exchange (4651 [49.7%] vs 107 834 [54.9%]; absolute difference, −5.3%; 95% CI, −6.3% to −4.2%) and had lower mean (SD) performance scores (22.8 [22.2] vs 25.5 [23.8]; absolute difference, −2.8; 95% CI, −3.5 to −2.1).

For nontechnology-dependent measures, psychiatrists reported at higher rates than other clinicians on 13 of 15 measures but had lower mean (SD) performance scores on 8 of 15 measures, including documentation of current medications (80.8 [20.1] vs 89.5 [14.1]; absolute difference, −8.7; 95% CI, −9.3 to −8.2), colorectal cancer screening (48.4 [19.7] vs 51.9 [20.0]; absolute difference, −3.4; 95% CI, −4.2 to 2.6), and breast cancer screening (53.7 [19.6] vs 57.1 [18.4]; absolute difference, −3.4; 95% CI, −4.2 to −2.6). However, among the measures that appear most relevant to the practice of psychiatry, psychiatrists had higher reporting rates and better performance, including for depression screening and follow-up (reporting rate, 2542 [27.2%] vs 36 157 [18.4%]; absolute difference, 8.8%; 95% CI, 7.9%-9.6%; mean [SD] performance score, 43.4 [28.5] vs 40.9 [27.7]; absolute difference, 2.5; 95% CI, 1.4-3.6), screening for future fall risk (reporting rate, 2599 [27.8%] vs 43 885 [22.3%]; absolute difference, 5.4%; 95% CI, 4.6%-6.3%; mean [SD] performance score, 63.2 [27.3] vs 58.4 [29.2]; absolute difference, 4.8; 95% CI, 3.7-6.0), and use of the PHQ-9 tool for depression screening (reporting rate, 1171 [12.5%] vs 17 062 [8.7%]; absolute difference, 3.8%; 95% CI, 3.2%-4.4%; mean [SD] total performance score, 26.1 [20.9] vs 24.8 [17.5]; absolute difference, 1.3; 95% CI, 0.3-2.4).

Finally, of the 25 MIPS measures included in the mental/behavioral health specialty (eTable 6 in the Supplement), we found that 13 measures were reported by less than 2% of psychiatrists, and 7 measures were not reported by any physicians in our sample.

## Discussion

In this cross-sectional study comparing psychiatrists with other outpatient physicians in the 2020 Medicare MIPS, psychiatrists had significantly lower performance scores and, consequently, were more likely to be penalized and less likely to receive bonus payments than their peers. These performance disparities were driven primarily by lower scores in the quality and promoting interoperability domains. In particular, psychiatrists performed more poorly on technology-dependent measures, such as participation in health information exchanges; care coordination measures, such as documentation of patient medications in medical records; and preventive care measures unrelated to psychiatry, such as cancer screening.

Taken together, our findings imply that psychiatrists may not be as well prepared as other outpatient physicians for the reporting and performance requirements of the MIPS program and may experience financial penalties as a result. As the size of value-based payment adjustments increases in MIPS in future years,^26^ in concert with increasing demand for psychiatric services from the aging Medicare population,^27^ psychiatric practitioners may experience significant financial implications. Many psychiatric practices already face low visit payment rates and narrow network restrictions^28,29,30,31^ in the Medicaid, Medicare Advantage, and commercial insurance markets. Psychiatrists are also substantially less likely to accept Medicare than any other nonpediatric physicians.^32,33,34,35^ The increased administrative and financial burdens introduced by MIPS may further disincentivize psychiatrists from treating Medicare patients,^33^ resulting in an even greater number of psychiatrists who require patients to pay out of pocket for services. This factor has concerning implications for access to mental health care for Medicare beneficiaries.

Our findings also raise additional questions about the relevance and appropriateness of the MIPS program for performance assessment of specialists.^5,6,7,8,9,10,11,12^ Ideally, each specialty would be judged on measures of greatest relevance to the patients treated by that specialty. For instance, within psychiatry, we would expect quality measures for evidence-based pharmaceutical treatment, appropriate referral to cognitive-behavioral therapy, and coordination and integration of services with primary care physicians.^36,37,38,39^ The fact that just as many psychiatrists in our exploratory analysis reported on quality measures for cancer screening and flu shots as for depression care suggests that MIPS performance reflects multispecialty group performance as opposed to quality of psychiatric care. Our finding that most measures included in the mental/behavioral health specialty set were almost entirely unreported indicates that further efforts are needed to develop and encourage use of measures relevant to psychiatric care.

Psychiatrists may also face greater challenges than other outpatient physicians because they are more likely to treat a caseload that includes a higher proportion of socially at-risk patients.^27^ We found that psychiatrists were more than twice as likely to treat patients dually enrolled in Medicaid and treat greater numbers of patients with disabilities or who belong to racial and ethnic minority groups. Prior research shows that Medicare clinicians with larger caseloads of patients with these characteristics perform worse on the MIPS and other value-based payment programs, perhaps because of inadequate risk adjustment for social risk factors.^40,41,42^ In addition, Medicare does not risk adjust for the most prevalent forms of depression and anxiety disorders, and prior research shows that this inadequate risk adjustment results in underestimation of the resources required to treat beneficiaries with these conditions.^42,43^ Treating patients with more social risk factors further increases the complexity of psychiatric visits and requires more resources for treatment, compounding the increased costs of caring for patients with mental health disorders.^17^ As a result, the CMS should track the effect of the MIPS program on the Part B participation rates of psychiatrists and other clinicians who disproportionately provide care for beneficiaries with social and mental health risk factors to ensure that basic levels of access to behavioral health care are maintained.

Whether the MIPS will meaningfully improve quality of psychiatric care for Medicare patients is unknown. Research on the first year of the MIPS program suggests that the quality-of-care measures reported to the CMS by physicians may be better explained by selective reporting behaviors designed to maximize reimbursement as opposed to actual quality of care.^9,25,44,45^ Longer-term studies are needed as future years of data become available to examine the effect of MIPS on psychiatry and behavioral health.

### Limitations

This study has several limitations. First, this study was dependent on publicly reported data from the CMS in the National Downloadable Files and MIPS data sets. The CMS did not publicly report MIPS performance data on low-volume MIPS-participating clinicians, and very low-volume Medicare physicians and those who participated in advanced APMs that shared financial risk with the CMS were excluded from the MIPS entirely. Second, we identified physicians by their unique NPIs, although the CMS identifies physicians in the MIPS by their unique NPI and tax identifier number combinations, counting the same physician multiple times. As a result, our participation counts are lower than those of the CMS. Third, this was an observational study; although the adjusted analysis controlled for multiple physician, caseload, and practice area factors, it is likely there is residual unmeasured confounding. More research is needed to uncover the causal mechanisms behind these findings.

## Conclusions

In this national cross-sectional study of Medicare psychiatrists and other outpatient physicians participating in the 2020 MIPS, psychiatrists received significantly lower performance scores, were penalized more frequently, and received fewer bonus payments than other outpatient physicians. The CMS may want to reconsider the use of many current MIPS measures for assessing the performance of psychiatrists.

## Notes

Supplement. **eTable 1.** Descriptive Statistics on Medicare-Serving Outpatient Physicians Eligible for Study but Excluded Due to Missing Data Compared to Physicians Included in Study Population **eTable 2.** Comparison of 2020 MIPS Participation Rates of Psychiatrists and Other Outpatient Physicians Listed in the National Downloadable File **eTable 3.** Comparison of Psychiatrists and Other Outpatient Physicians Participating in the 2020 MIPS on Additional Patient Characteristics in the Medicare Physician and Other Supplier Reports Database **eTable 4.** Sensitivity Analyses for Association of Psychiatry Versus Other Outpatient Physician Specialty Type with 2020 Merit-Based Incentive Payment System Performance Scores **eTable 5.** Association of Psychiatry Versus Other Outpatient Physician Specialty Type with 2020 Merit-Based Incentive Payment System Performance Domain Scores **eTable 6.** Reporting Frequency of Merit-Based Incentive Payment System Performance Measures in the Mental/Behavioral Health Specialty Set
