# Supplementary material for: Comparison of Performance of Psychiatrists vs Other Outpatient Physicians in the 2020 US Medicare Merit-Based Incentive Payment System
Source: JAMA Health Forum. 2022 Mar 25;3(3):e220212. doi: 10.1001/jamahealthforum.2022.0212 (PMC8956979; doi:10.1001/jamahealthforum.2022.0212)
Supplement: Supplement. — eTable 1. Descriptive Statistics on Medicare-Serving Outpatient Physicians Eligible for Study but Excluded Due to Missing Data Compared to Physicians Included in Study Population eTable 2. Comparison of 2020 MIPS Participation Rates of Psychiatrists and Other Outpatient Physicians Listed in the National Downloadable File eTable 3. Comparison of Psychiatrists and Other Outpatient Physicians Participating in the 2020 MIPS on Additional Patient Characteristics in the Medicare Physician and Other Supplier Reports Database eTable 4. Sensitivity Analyses for Association of Psychiatry Versus Other Outpatient Physician Specialty Type with 2020 Merit-Based Incentive Payment System Performance Scores eTable 5. Association of Psychiatry Versus Other Outpatient Physician Specialty Type with 2020 Merit-Based Incentive Payment System Performance Domain Scores eTable 6. Reporting Frequency of Merit-Based Incentive Payment System Performance Measures in the Mental/Behavioral Health Specialty Set [file jamahealthforum-e220212-s001.pdf]

Supplementary Online Content

Qi AC, Joynt Maddox KE, Bierut LJ, Johnston KJ. Comparison of performance of psychiatrists vs other outpatient physicians in the 2020 US Medicare Merit-Based Incentive Payment System. *JAMA Health Forum.* 2022;3(3):e220212. doi:10.1001/jamahealthforum.2022.0212

eTable 1. Descriptive Statistics on Medicare-Serving Outpatient Physicians Eligible for Study but Excluded Due to Missing Data Compared to Physicians Included in Study Population

eTable 2. Comparison of 2020 MIPS Participation Rates of Psychiatrists and Other Outpatient Physicians Listed in the National Downloadable File

eTable 3. Comparison of Psychiatrists and Other Outpatient Physicians Participating in the 2020 MIPS on Additional Patient Characteristics in the Medicare Physician and Other Supplier Reports Database

eTable 4. Sensitivity Analyses for Association of Psychiatry Versus Other Outpatient Physician Specialty Type with 2020 Merit-Based Incentive Payment System Performance Scores

eTable 5. Association of Psychiatry Versus Other Outpatient Physician Specialty Type with 2020 Merit-Based Incentive Payment System Performance Domain Scores

eTable 6. Reporting Frequency of Merit-Based Incentive Payment System Performance Measures in the Mental/Behavioral Health Specialty Set

This supplementary material has been provided by the authors to give readers additional information about their work.

© 2022 Qi AC et al. *JAMA Health Forum*.

eTable 1: Descriptive Statistics on Medicare-Serving Outpatient Physicians Eligible for Study But Excluded Due to Missing Data Compared to Physicians Included in Study Population.

	Excluded From Study Population[a]			Study Population[b]	
**Total Number of Physicians**	**26,718**			**205,662**	
**Physician Specialty Type**	**N**			**N**	
Psychiatrist	26,718	9%		205,662	5%
Other Outpatient Physician		91%			95%
**2018 MIPS Performance**	**N**			**N**	
Final Score (0-100)	26718	92		205,662	89
Negative Payment Adjustment		1%			3%
Positive Payment Adjustment		99%			96%
Bonus Payment Adjustment		93%			88%
**2018 MIPS Reporting Classification**					
Individual	26718	3%		205,662	96%
Group		50%			88%
Alternative Payment Model		46%			48%
**Clinician Characteristics**					
Gender					
Female	26,718	46%		205,662	35%
Male		54%			65%
Years Since Medical School Graduation, *MEAN*	25,739	21		205,662	23
Primary Care Physician[c]	26,718	43%		205,662	51%
Specialist[d]		57%			49%
Affiliated with a Safety-Net Hospital	26,718	33%		205,662	33%
Affiliated with a Major Teaching Hospital[f]	26,718	39%		205,662	27%
**Treated Medicare Beneficiaries' Characteristics**					
Total Medicare Beneficiaries, *MEAN*	13,861	290		205,662	425
Medicare-Medicaid Dually Eligibles, %	11,649	34%		178,014	28%
Average Age, *MEAN*	13,861	68.4		205,662	71.2
Gender					
Female	12,872	57%		198,287	59%
Male		43%			41%
Race					
White	10,471	71%		168,147	78%
Nonwhite		29%			22%
CMS-HCC Risk Score, *MEAN*	13,861	2.15		205,662	1.78

© 2022 Qi AC et al. *JAMA Health Forum*.

**Local Practice Area Characteristics[h]**					
Area Deprivation Index National Rank,[i] *MEAN*	12,183	43		205,662	45
Rural[k]	25,181	8%		205,662	16%
Urban[k]		93%			90%
Census Region					
Northeast	26,718	25%		205,662	22%
Midwest		34%			37%
South		21%			22%
West		19%			18%

Abbreviations: N, number; MIPS, Merit-Based Incentive Payment System; CMS, Centers for Medicare and Medicaid Services; HCC, Hierarchical Condition Categories.

[a]Met study inclusion criteria due to receiving a publicly-reported MIPS 2020 score on Physician Compare and included in Physician Compare National Downloadable File between 2018-2020 with specialty type of primary care, medical specialty, obstetrics/gynecology, or psychiatry, but excluded from study due to no records in the other study databases or missing data on any of the study variables.

[b]Study inclusion criteria: received a publicly-reported MIPS score for 2020 in the Provider Data Catalog (formerly Physician Compare) and included in the National Downloadable File between 2018-2020 with specialty type of primary care, medical specialty, obstetrics/gynecology, or psychiatry. Excluded clinicians with no records in the following databases: 1) 2018 Medicare Physician and Other Supplier Reports; 2) 2015 geocoded US Census Block Group data; 3) 2018 Area Deprivation Index Census Block Group data; 4) 2010 Census Tract Rural-Urban Commuting Area codes; 5) 2018 Dartmouth Atlas of Healthcare Hospital Referral Region ZIP Code Crosswalk. Further excluded clinicians missing data on any of the study variables above.

[c]Primary care physician includes all physicians who listed a medical specialty of geriatric medicine, internal medicine, family medicine, general practice, obstetrics/gynecology, or pediatric medicine.

[d]Specialist includes all physicians who were not primary care physicians.

[e]Safety-Net Provider defined as a physician with a safety-net hospital affiliation (identified by hospital affiliation in Physician Compare linked to hospital DSH percent in the top quartile in the 2018 CMS Impact File).

[f]Identified by hospital affiliation in Physician Compare linked to a Major Teaching Hospital, defined as a general acute care hospital with a resident-to-bed ratio of ≥0.25 in the 2018 CMS Impact File.

[h]Note that many physicians have multiple practice locations. As a result, the Area Deprivation Index National Rank represents that average for each physician across all of their practice locations. In addition, some physicians had practice locations in both rural and urban areas, and others had practice locations in more than one Census Region; thus these numbers do not sum to 100%.

[i]The Area Deprivation Index is a measure of local neighborhood area socioeconomic disadvantage derived from Census Tract data on income, education, employment, and housing quality. The Area Deprivation Index National Rank ranges from 0 to 100 and indicates the percentile rank of disadvantage for a given Census Tract. Numbers closer to 100 indicate greater disadvantage. Numbers close to 50 (as above) are indicative of the national average.

[k]Physicians with at least one practice location in a rural or urban census tract by RUCA code are counted under the respective classification.

© 2022 Qi AC et al. *JAMA Health Forum*.

eTable 2. Comparison of 2020 MIPS Participation Rates of Psychiatrists and Other Outpatient Physicians Listed in the National Downloadable File.

	All Clinicians			Psychiatrist		Other Outpatient Physicians		P-Value[b]
**Total Number of Physicians[a]**	**N = 402,602**			**N = 28,522**		**N = 374,080**		**--**
	**N**			**N**		**N**		
Number of Physicians Participating, %	232,848	58%		11,730	41%	221,118	59%	<0.001
Number of Physicians Not Participating, %	169,754	42%		16,792	59%	152,962	41%	

Abbreviations: N, number; MIPS, Merit-based Incentive Payment System

[a]Includes all physicians who billed Medicare between 2018-2020 and hence listed in the National Downloadable File for this period with specialty type of primary care, medical specialty, obstetrics/gynecology, or psychiatry.

[b]P-values for difference in proportions (Chi-square test) across psychiatrist versus non-psychiatrist specialty type.

© 2022 Qi AC et al. *JAMA Health Forum*.

eTable 3: Comparison of Psychiatrists and Other Outpatient Physicians Participating in the 2020 MIPS on Additional Patient Characteristics in the Medicare Physician and Other Supplier Reports Database.

	**All Clinicians**			**Psychiatrist**		**Other Outpatient Physicians**		**P-Value[b]**
**Total Number of Physicians[a]**	**N = 205,662**			**N = 9,356**		**N = 196,306**		**--**
	**N**			**N**		**N**		
Patients Diagnosed with Depression, %	193,335	32%		9,016	69%	184,319	30%	<0.001
Medicare-Medicaid Dually Eligibles, %	178,014	28%		7,350	57%	170,664	27%	<0.001
Average Age, *MEAN*	205,662	71.2		9,356	59.6	196,306	71.8	<0.001
Gender								
Female	198,287	59%		8,249	55%	190,038	59%	<0.001
Male		41%			45%		41%	
Race								
White	168,147	78%		6,374	73%	161,773	78%	<0.001
Nonwhite		22%			27%		22%	

Abbreviations: N, number; MIPS, Merit-based Incentive Payment System

[a]Study inclusion criteria: received a publicly-reported MIPS score for 2020 in the Provider Data Catalog (formerly Physician Compare) and included in the National Downloadable File between 2018-2020 with specialty type of primary care, medical specialty, obstetrics/gynecology, or psychiatry. Excluded clinicians with no records in the following databases: 1) 2018 Medicare Physician and Other Supplier Reports; 2) 2015 geocoded US Census Block Group data; 3) 2018 Area Deprivation Index Census Block Group data; 4) 2010 Census Tract Rural-Urban Commuting Area codes; 5) 2018 Dartmouth Atlas of Healthcare Hospital Referral Region ZIP Code Crosswalk. Further excluded clinicians missing data on any of the study variables above.

[b]P-values for difference in proportions (Chi-square test) or means (independent sample two-tailed t-test) across psychiatrist versus non-psychiatrist specialty type.

© 2022 Qi AC et al. *JAMA Health Forum*.

eTable 4. Sensitivity Analyses for Association of Psychiatry Versus Other Outpatient Physician Specialty Type with 2020 Merit-Based Incentive Payment System Performance Scores.

		**Expanded Population: Adjustment for Fewer Beneficiary Characteristics[a]**			**Reduced Population: Adjustment for Additional Beneficiary Characteristics[b]**	
		**Marginal Effect**	**95% Confidence Interval**		**Marginal Effect**	**95% Confidence Interval**
**Total Number of Physicians**		**N = 232,380**			**N = 154,385**	
Final Performance Score		-4.0	(-4.5, -3.6)		-9.1	(-9.9, -8.4)
Negative Payment Adjustment, %		1.6	(1.3, 1.9)		4.0	(3.3, 4.7)
Positive Payment Adjustment, %		-1.8	(-2.1, -1.5)		-5.1	(-5.9, -4.3)
Bonus Payment Adjustment, %		-4.6	(-5.2, -3.9)		-10.0	(-11.3, -8.7)
Abbreviations: N, number; MIPS, Merit-based Incentive Payment System.						

[a]Expanded study population includes all physicians with a publicly-reported MIPS score for 2020 and included in the National Downloadable File between 2018-2020 with specialty type of primary care, medical specialty, obstetrics/gynecology, or psychiatry. We estimated 4 multivariable regression models: a) with ordinary least squares and Final Performance Score as the dependent variable or b-d) with logistic regression and the three Payment Adjustment indicators as the dependent variables. In all 4 models, an indicator for psychiatrist specialist type was the independent variable with physician gender, number of years since medical school graduation, affiliation with a safety-net hospital, and affiliation with a major teaching hospital as control variables. We report the marginal difference in the outcome as the change in the mean of the dependent variables associated with a unit change in the independent variables (i.e., the marginal effect).

[b]Study population from Table 2 further reduced to physicians who also had data from the Medicare Supplier and Other Reports database on percent of treated patients dually enrolled in Medicare and Medicaid, average patient age, percent of patients who were male and female, and percent of patients who were white and nonwhite. We estimated 4 multivariable regression models as in the primary analysis in Table 2. We report the marginal difference in the outcome as the change in the mean of the dependent variables associated with a unit change in the independent variables (i.e., the marginal effect).

© 2022 Qi AC et al. *JAMA Health Forum*.

eTable 5. Association of Psychiatry Versus Other Outpatient Physician Specialty Type with 2020 Merit-Based Incentive Payment System Performance Domain Scores.

		Unadjusted Results				Adjusted Means[a]		
		**Psychiatrist**	**Non-Psychiatrist**	**Absolute Difference (95% CI)**		**Psychiatrist**	**Non-Psychiatrist**	**P-Value[b]**
**Total Number of Clinicians**		**9,356**	**196,306**	**--**		**--**	**--**	**--**
**Clinicians Reporting Quality Score**		**99%**	**99%**					
Quality Score, *MEAN (SD)*		80.3 (32.9)	86.7 (26.6)	-6.3 (-6.1, -5.1)		79.6	86.7	<0.001
**Clinicians Reporting Promoting Interoperability Score**		**91%**	**93%**					
Promoting Interoperability Score, *MEAN (SD)*		84.1 (34.9)	90.0 (27.3)	-5.9 (-6.1, -5.1)		83.5	90.1	<0.001
**Clinicians Reporting Improvement Activities Score**		**100%**	**100%**					
Improvement Activities Score, *MEAN (SD)*		36.4 (11.3)	37.6 (9.3)	-1.2 (-6.1, -5.1)		36.1	37.6	<0.001
**Clinicians Reporting Cost Score**		**34%**	**40%**					
Cost Score, *MEAN (SD)*		71.5 (18.8)	72.5 (18.5)	-1.0 (-6.1, -5.1)		71.0	72.5	<0.001
Abbreviations: SD, standard deviation; CI, confidence interval.								

[a]We estimated a multivariable regression model that also adjusted for the individual clinician, Medicare patient caseload, and local practice area characteristics listed in Table 1. We used ordinary least squares regression to model the final performance score outcome and individual domain scores.

[b]We report the adjusted means of the scores with Wald testing of significance between psychiatrist and other outpatient physicians means.

© 2022 Qi AC et al. *JAMA Health Forum*.

eTable 6. Reporting Frequency of Merit-Based Incentive Payment System Performance Measures in the Mental/Behavioral Health Specialty Set.

	Percent Reporting[a]	
	**All Physicians**	**Psychiatrists**
**Total Number of Physicians in Study**	**205,662**	**9,356**
**Mental/Behavioral Health Specialty Set Measures**		
Preventive Care and Screening: Body Mass Index (BMI) Screening and Follow-Up Plan	26.6	33.7
Documentation of Current Medications in the Medical Record	25.5	28.3
Preventive Care and Screening: Screening for Depression and Follow-Up Plan	18.8	27.2
Preventive Care and Screening: Tobacco Use: Screening and Cessation Intervention	20.2	25.4
Depression Utilization of the PHQ-9 Tool	8.9	12.5
Adult Major Depressive Disorder (MDD): Suicide Risk Assessment	3.0	7.5
Depression Remission at Twelve Months	4.3	7.4
Closing the Referral Loop: Receipt of Specialist Report	6.7	7.2
Anti-Depressant Medication Management	3.1	7.0
Child and Adolescent Major Depressive Disorder (MDD): Suicide Risk Assessment	2.2	6.3
Dementia: Cognitive Assessment	4.8	5.6
Preventive Care and Screening: Unhealthy Alcohol Use: Screening & Brief Counseling	1.7	3.9
Bipolar Disorder and Major Depression: Appraisal for alcohol or chemical substance use	1.0	1.9
Tobacco Use and Help with Quitting Among Adolescents	1.7	1.7
Dementia Associated Behavioral and Psychiatric Symptoms Screening and Management	0.1	0.4
Dementia: Safety Concerns Screening and Mitigation Recommendations or Referral for Patients with Dementia	0.1	0.2
Elder Maltreatment Screen and Follow-Up Plan	0.1	0.2
Dementia: Functional Status Assessment	0.1	0.2
Adherence to Antipsychotic Medications For Individuals with Schizophrenia	0.0	0.0
Adult Major Depressive Disorder (MDD): Coordination of Care of Patients with Specific Comorbid Conditions	0.0	0.0
Dementia: Caregiver Education and Support	0.0	0.0
Depression Remission at Six Months	0.0	0.0
Follow-Up After Hospitalization for Mental Illness (FUH)	0.0	0.0
Follow-Up Care for Children Prescribed ADHD Medication (ADD)	0.0	0.0
Preventive Care and Screening: Screening for High Blood Pressure and Follow-Up Documented	0.0	0.0
Abbreviations: N, number; MIPS, Merit-Based Incentive Payment System.		
[a]Physicians who met our study inclusion and exclusion criteria and had data publicly reported on individual MIPS measures. Note that not all physicians included in our study had data publicly reported on individual measures (although they did all have an overall MIPS score and payment adjustment reported).		

© 2022 Qi AC et al. *JAMA Health Forum*.
